# Measuring the frequency and variation of unnecessary care across Canada

**DOI:** 10.1186/s12913-019-4277-9

**Published:** 2019-07-03

**Authors:** Zachary Bouck, Ciara Pendrith, Xi-Kuan Chen, Jennifer Frood, Ben Reason, Tanya Khan, Alicia Costante, Kyle Kirkham, Karen Born, Wendy Levinson, R. Sacha Bhatia

**Affiliations:** 1 Institute for Health Systems Solutions and Virtual Care, Women’s College Hospital, 76 Grenville Street, 6th Floor, Toronto, Ontario M5S 1B2 Canada; 2 Cumming School of Medicine, University of Calgary, 3330 Hospital Drive NW, Calgary, Alberta T2N 4N1 Canada; 3 Canadian Institute for Health Information, 4110 Yonge Street, Suite 300, Toronto, Ontario M2P 2B7 Canada; 4 Canadian Institute for Health Information, 1010 Sherbrooke Street West, Suite 602, Montreal, Quebec H3A 2R7 Canada; 5 Department of Anesthesia, Women’s College Hospital, 76 Grenville Street, Toronto, Ontario M5S 1B2 Canada; 6 Choosing Wisely Canada, 250 Yonge Street, Room 648, Toronto, Ontario M5G 1B1 Canada; 7 Department of Medicine, University of Toronto – St Michael’s Hospital, 30 Bond Street, Toronto, Ontario M5B 1W8 Canada

**Keywords:** Choosing wisely, Low-value, Lower back pain, Preoperative testing, Mammography

## Abstract

**Background:**

Through the Choosing Wisely Canada (CWC) campaign, national medical specialty societies have released hundreds of recommendations against health care services that are unnecessary, i.e. present little to no benefit or cause avoidable harm. Despite growing interest in unnecessary care both within Canada and internationally, prior research has typically avoided taking a national or even multi-jurisdictional approach in measuring the extent of the issue. This study estimates use of three unnecessary services identified by CWC recommendations across multiple Canadian jurisdictions.

**Methods:**

Two retrospective cohort studies were conducted using administrative health care data collected between fiscal years 2011/12 and 2012/13 to respectively quantify use of 1) diagnostic imaging (spinal X-ray, CT or MRI) among Albertan patients following a visit for lower back pain and 2) cardiac tests (electrocardiogram, chest X-ray, stress test, or transthoracic echocardiogram) prior to low-risk surgical procedures in Alberta, Saskatchewan, and Ontario. A cross-sectional study of the 2012 Canadian Community Health Survey was also conducted to estimate 3) the proportion of females aged 40–49 that reported having a routine mammogram in the past two years.

**Results:**

Use of unnecessary care was relatively frequent across all three services and jurisdiction measured: 30.7% of Albertan patients had diagnostic imaging within six months of their initial visit for lower back pain; a cardiac test preceded 17.9 to 35.5% of low-risk surgical procedures across Alberta, Saskatchewan, and Ontario; and 22.2% of Canadian women aged 40–49 at average-risk for breast cancer reported having a routine screening mammogram in the past two years.

**Conclusions:**

The use of potentially unnecessary care appears to be common in Canada. This investigation provides methodology to facilitate future measurement efforts that may incorporate additional jurisdictions and/or unnecessary services.

**Electronic supplementary material:**

The online version of this article (10.1186/s12913-019-4277-9) contains supplementary material, which is available to authorized users.

## Background

The Institute of Medicine (IOM) estimated in 2012 that 30% of annual health care spending is wasteful, citing unnecessary services and inefficient care as primary drivers of cost [1]. Months before the IOM report, Choosing Wisely (CW) launched in the United States [2]. CW is a clinician-led campaign with the intention of encouraging clinicians and patients to engage in conversations about potentially unnecessary services – i.e. tests, treatments, and procedures where care is unlikely to be beneficial and/or may cause avoidable harm to the patient [2–4]. Since the launch of CW in 2012, the CW campaign has been adopted internationally with over 20 countries that have either started their own versions of the campaign or have local CW campaigns in development [4]. As interest in tackling the problem of unnecessary care grows worldwide, so does the need for quantifying the extent of the issue at a national level [4]. Initial estimates of the prevalence of unnecessary care are predominantly derived from U.S. data [5]; however, prior studies from the U.S. have largely been limited to Medicare beneficiaries or select commercial health plan members [6–8]. While some population-based studies have assessed overuse in Australia and Canada, these works have often been limited to a single region [9–13]. To our knowledge, there has not been a prior study that quantifies the use of unnecessary care across multiple services and Canadian jurisdictions.

Based on a published framework for measuring unnecessary or “low-value” care, we aim to develop common methodological approaches to estimating overuse at a national level by utilizing existing, routinely collected administrative and survey data in Canada [14]. To facilitate measurement across multiple Canadian jurisdictions, the Choosing Wisely Canada (CWC) campaign partnered with the Canadian Institute for Health Information (CIHI) to provide large-scale estimates of unnecessary care across provinces and territories. By piloting a practical application of this measurement framework, we hope to develop standardized methods of measuring unnecessary care that might be applied across Canada and, in doing so, may serve as a model for how other countries approach quantifying unnecessary care at a national level [14].

In this paper, we focus on three CWC recommendations relevant to primary care that advise against the following potentially unnecessary tests: 1) imaging for lower back pain in the absence of red flags [15, 16]; 2) routine performance of preoperative testing for patients undergoing low-risk surgeries [17–19]; and 3) routine screening mammography for average-risk women aged 40–49 [16, 20]. These three CWC recommendations, highlighted in CIHI’s recent *Unnecessary Care in Canada* report [21], were identified by multiple Canadian specialty societies to be of concern and were suspected to be common practice despite evidence-based recommendations against their use [15–20]. The objectives of this study are to quantify the frequency of these unnecessary screening and diagnostic imaging services across multiple Canadian provinces, and to investigate factors that may affect their likelihood of being ordered.

## Methods

### Study design, data sources, and analysis

We conducted three separate studies to measure the use of services identified in each of the CWC recommendations chosen. The following section summarizes the study designs, data sources, and other methodological considerations for investigating each recommendation (Additional file 1: Table S1, Additional file 2: Table S2, Additional file 3: Table S3 and Additional file 4: Table S4 detail complete methodology). All data, irrespective of source, was collected between 2011 and 2013 to assess the prevalence of overuse in Canada prior to the introduction of CWC. CIHI is an independent not-for-profit organization that has been established to collect and report on health outcomes across Canada. CIHI is a prescribed entity under section 45 [1] of Ontario’s Personal Health Information Protection Act, allowing CIHI to hold personal health information for the purposes of compiling statistical information for the management of the health system [22]. The use of routinely collected data – either during patient care (CIHI administrative data) or at set cycles (Health Canada surveys) – may enable future measurement and comparison with the baseline estimates contained herein, possibly to evaluate the impact of the CWC campaign or other interventions implemented after 2013 to decrease low-value care. As this study used routinely collected data that was analyzed at CIHI, in accordance with their institutional privacy policies, this study was exempted from research ethics approval.

Analysis followed the same general approach per recommendation: 1) estimate the frequency of, and variation in, the use of unnecessary care service(s) identified in that recommendation and 2) conduct regression-based analyses to identify factors associated with use of those services. Recommendation-specific methodology is outlined in the following sections, including variable selection for our multiple logistic regression analyses. Missing data was handled via list-wise deletion. All analyses were performed using SAS version 9.4 and statistical significance was assessed at a two-tailed *P* ≤ .05.

### Lower back pain imaging

An Ontario-based study informed measurement of lower back pain imaging in Alberta [11]. Our investigation was limited to Alberta for this recommendation, as the study required community physician, hospital, and clinic data that CIHI only had access to in this province. Visits to family physicians for lower pain back by Albertan patients aged 18 and older between April 1st, 2011 and March 31st, 2012 were identified using International Classification of Diseases (ICD) 9th revision (ICD-9) coded claims in the Patient-Level Physician Billing (PLPB) Data Repository [11]. We excluded patients with any ‘red flag’ condition(s) (e.g. cancer, vertebral compression fracture, neurological problems) that may warrant investigation with lower back pain imaging within one year prior to the index visit – identified via ICD-9 codes in the PLPB or ICD 10th revision (ICD-10) codes in National Ambulatory Care Reporting System (NACRS) and Discharge Abstract Database (DAD) claims [11]. To limit the cohort to patients with non-persistent lower back pain, we excluded patients with prior visits, admissions, surgical procedures or diagnostic imaging for lower back pain within one year of the index visit via ICD-9, ICD-10 and Canadian Classification of Health Interventions (CCI) codes across NACRS, DAD, and PLPB claims data (see Additional file 2: Table S2 for complete definitions). For each patient meeting these criteria, only their first qualifying visit was included.

The primary outcome was receipt of any lower back imaging (X-ray or CT/MRI) at six months post-visit [23]. Spinal X-rays were identified from the NACRS and PLPB using Canadian Classification of Health Interventions (CCI) codes and fee codes respectively. Receipt of computed tomography (CT) or magnetic resonance imaging (MRI) claims were identified using CCI-coded NACRS data. CT and MRI claims were considered a single outcome due to their common decision-making pathways. The unadjusted diagnostic imaging rate by time from index visit and test type (X-ray or CT/MRI) was observed. Follow-up at six months was informed by Albertan wait time data for related diagnostic imaging [24].

For each eligible lower back pain visit, we captured several characteristics for both patient (age, sex, neighbourhood income quintile, and rurality) and physician (medical specialty [general practitioner or specialist], compensation model [fee-for-service or other], and years in practice) previously associated with the use of potentially unnecessary imaging [11, 25]. Neighbourhood income quintile was determined based on patient’s postal code to approximate their socioeconomic status [26]. Additionally, patients with history of any of the following comorbidities in the past three years were identified: coronary artery disease, congestive heart failure, atrial fibrillation, other cardiac arrhythmia, cardiac valvular disease, renal disease, previous cardiovascular disease, peripheral vascular disease, venous thromboembolism, COPD, diabetes, hypertension; and asthma. Prior studies have suggested that physicians who see a larger volume of patients with a specific condition (e.g., lower back pain) may provide higher quality care for those patients [27]. Correspondingly, we also measured the number of lower back pain patients seen per physician per year. Multivariable logistic regression was conducted to estimate the relationship between patient and physician characteristics with the odds of having lower back imaging (X-ray or CT/MRI), expressed via odds ratios (OR) with corresponding 95% confidence intervals (CI). Generalized estimation equations were used in estimating model parameters to account for possible correlation within physicians. Imaging rates of the primary outcome were then adjusted for all patient and physician characteristics found to be significant in the primary regression model and reported by Albertan health zone.

### Preoperative cardiac testing

As with lower back pain, a prior Ontarian study informed measurement of preoperative cardiac testing [10]. The Institute of Clinical Evaluative Sciences (ICES) shared data from the corresponding study to facilitate comparison of Ontarian rates with additional provinces. The comparability of billing codes resulted in patients from Ontario, Alberta, and Saskatchewan being included.

Using methodology published by Kirkham and colleagues, low-risk surgical procedures were defined as procedures with an estimated risk of myocardial infarction or cardiac-related death of < 1% and where preoperative testing is typically not required [10]. Eligible procedures were identified via CCI procedural codes as primary interventions either performed in an ambulatory care setting or occurring in acute care on the same day as admission [10]. Procedure type was classified as endoscopic, ophthalmologic, or other (e.g. knee arthroscopy, hernia repair) [10].

Any receipt of a preoperative cardiac test – electrocardiogram (ECG), transthoracic echocardiogram (TTE), stress test, or chest X-ray – within 60 days of an eligible surgical procedure was recorded between June 2012 and March 2013 via provincial billing and CCI codes. Duplicate tests were identified by linking health card number, test location and type across datasets and subsequently excluded. Tests occurring on the same date as surgery may have been postoperative and were excluded (Additional file 3: Table S3).

Additional exclusion criteria were applied to examine preoperative cardiac testing rates by individual facilities and providers. Facilities with less than 50 low-risk procedures performed between June 2012 and March 2013 were excluded [10]. Among facilities satisfying the preceding criteria, provider-level ordering rates were calculated for physicians who performed at least 25 low-risk operations [10]. Ontario was excluded from these investigations as the data shared by ICES was reported at the provincial level.

Multiple logistic regression models were created to model the odds of a cardiac test preceding a low-risk surgery in Alberta and Saskatchewan separately. Receipt of any of the four tests was aggregated into a single outcome. For each province, two logistic models were regressed (both adjusting for procedure type) to independently investigate patient- and provider-level factors previously associated with use of low-value preoperative tests [9, 10]. Specifically, we captured patient characteristics (age, sex, and history of the same comorbidities listed in the lower back pain study), surgical procedure type, and whether the patient received a pre-operative consult (i.e., general medical or anesthetic) [10].

### Screening mammography

The Canadian Community Health Survey (CCHS) was used to investigate the prevalence of screening mammography among women aged 40–49 despite average-risk status [16, 20]. The 2012 CCHS was selected as, at the time of analysis, it was the most recent CCHS survey to include a mammography module [28, 29]. The CCHS is representative of approximately 97% of the Canadian population and excludes individuals living on Aboriginal reserves, full-time members of the Canadian Forces, institutionalized populations (e.g. long-term care residents, prisoners) and individuals living in select remote Quebec health regions [28]. To adhere to Statistics Canada’s rules for CCHS Public Use Microdata File release, survey weights and conditional rounding were applied to all analyses (Additional file 4: Table S4) [29].

Breast cancer risk was approximated by female respondents’ answers to select CCHS questions. The study population for the primary analysis consisted of all female respondents aged 40–49, irrespective of risk status. Among these women, the number of respondents who reported a mammogram in the past two years and cited age and/or routine screening as the only reason(s) for the test qualified as average-risk (Additional file 4: Table S4). This definition was developed by CIHI with input from the CWC family medicine group.

Unadjusted proportions of routine mammogram screening among women 40–49 years old were reported both nationally and, wherever possible, by province or territory (i.e. where the number of unweighted respondents ≥30 and coefficient of variation ≤33.3) [29]. The results of a 2011 environmental scan by the Canadian Partnership Against Cancer on jurisdictional screening mammography guidelines – subsequently validated by members of the Canadian Breast Cancer Screening Network – were used to determine whether women aged 40–49 were eligible (by physician referral or self-referral) or ineligible for screening by province/territory [30].

Initially, a multivariable logistic regression was fit to model the odds of self-reporting screening mammogram in the past two years with adjustment for all of the following patient characteristics: marital status; total household income; physical activity; BMI group; education level; self-perceived health; type of smoker; cultural or racial origin; time in Canada; having a regular medical doctor; and receipt of Pap test in last three years [31–33]. Additionally, we identified each respondent’s eligibility for breast cancer screening based on their age and jurisdiction (i.e., eligible with self-referral, eligible with physician referral, or not eligible) [30]. Only characteristics with a corresponding *P*-value≤.15 were retained in the final model. To model the odds of an average-risk woman having a screening mammogram, any respondents who indicated a mammogram for a high-risk or symptomatic reason were excluded from regression analysis.

## Results

### Lower back pain imaging

The resulting cohort consisted of 97,740 adult Albertans who made their first visit to a primary care physician for non-persistent lower back pain in 2011/2012. Overall, 30.7% of patients had potentially unnecessary imaging (X-ray and/or CT/MRI) within six months of their initial visit despite the absence of red flags. Most tests were X-rays (29.1%) with only 4.6% receiving a CT/MRI.

Table 1 shows the adjusted associations of patient- and provider-level characteristics with receipt of imaging (spinal X-ray or CT/MRI) at six months. Having an X-ray was associated with older age (85+ v 18–44, odds ratio [OR] 1.98, *P* < .001; 65–84 v 18–44, OR 1.92, *P* < .001; and 45–64 v 18–44, OR 1.37, *P* < .001), being male (female v male, OR 0.96, *P* = .013) and belonging to the highest versus lowest income quintile (OR 1.05, *P* = .024). Similar associations were observed for receipt of a follow-up CT or MRI. Compared to patients living in urban areas, patients residing in rural areas had significantly greater odds of having a CT/MRI (OR 1.38, *P* < .001). Having a doctor with a low volume of lower back pain patients (i.e. < 50 per year) was associated with increased odds of having an X-ray (OR 1.16, *P* < .001) or CT/MRI (OR 1.44, *P* < .001).

**Table 1:** Association between patient- and physician-level characteristics and having a spinal X-ray or CT/MRI within 6 months after an index visit for non-persistent lower back pain in Alberta between April 1st, 2011 and March 31st, 2012 (*N* = 97,740)

		X-ray		CT/MRI	
Characteristic	n	Rate per 100^a^	Odds Ratio (95% CI)^b^	Rate per 100^a^	Odds Ratio (95% CI)^b^
*Patient-level*					
Age					
18–44 [ref]	49,623	24.6	1.00	3.2	1.00
45–64	35,736	31.7	1.37 (1.32–1.41)*	5.8	1.66 (1.55–1.77)*
65–84	11,063	39.8	1.92 (1.83–2.01)*	6.7	1.85 (1.69–2.02)*
85+	1318	39.9	1.98 (1.76–2.22)*	3.6	0.97 (0.71–1.31)
Sex					
Male [ref]	46,167	29.2	1.00	5.0	1.00
Female	51,573	29.0	0.96 (0.94–0.99)*	4.2	0.81 (0.76–0.86)*
Income Quintile					
1st – lowest [ref]	21,598	28.6	1.00	3.7	1.00
2nd	21,028	29.1	1.02 (0.98–1.06)	4.4	1.13 (1.03–1.24)*
3rd	19,732	29.3	1.03 (0.98–1.07)	4.7	1.15 (1.05–1.26)*
4th	18,969	29.2	1.03 (0.99–1.08)	4.9	1.19 (1.09–1.31)*
5th – highest	15,625	30.2	1.05 (1.01–1.10)*	5.3	1.25 (1.14–1.38)*
Rurality					
Urban [ref]	80,951	28.7	1.00	4.1	1.00
Rural	16,275	31.6	1.05 (0.99–1.10)	7.0	1.38 (1.27–1.49)*
*Physician-level*					
Annual LBP patient volume					
50+ [ref]	54,978	27.2	1.00	3.6	1.00
50	42,762	31.6	1.16 (1.09–1.22)*	5.8	1.44 (1.30–1.58)*
Specialty					
Specialist [ref]	1018	27.7	1.00	10.3	1.00
General practitioner	96,722	29.1	1.19 (0.97–1.47)	4.5	0.53 (0.41–0.70)*
Compensation model					
Non-FFS	1878	28.8	1.00	6.1	1.00
FFS	95,862	29.1	1.21 (1.06–1.37)*	4.5	1.02 (0.81–1.29)

Note: * P ≤ .05; CT/MRI = having either a CT or MRI; CI = confidence interval; LBP = lower back pain; FFS = fee-for-service; [ref] = reference category

^a^rate is the observed imaging rate (# tests/n) at index visit + 6 months for each patient in denominator

^b^obtained via multivariable regression with adjustments for all patient and physician characteristics listed in table (in addition to patient comorbidity indicator and physician years in practice - both statistically insignificant) using GEEs to account for possible clustering within physicians. Patient comorbidity indicator represented whether patient had history of any of the following comorbidities: coronary artery disease, congestive heart failure, atrial fibrillation, other cardiac arrhythmia, cardiac valvular disease, renal disease, previous cardiovascular disease, peripheral vascular disease, venous thromboembolism, COPD, diabetes, hypertension, and/or asthma

**Fig. 1:**
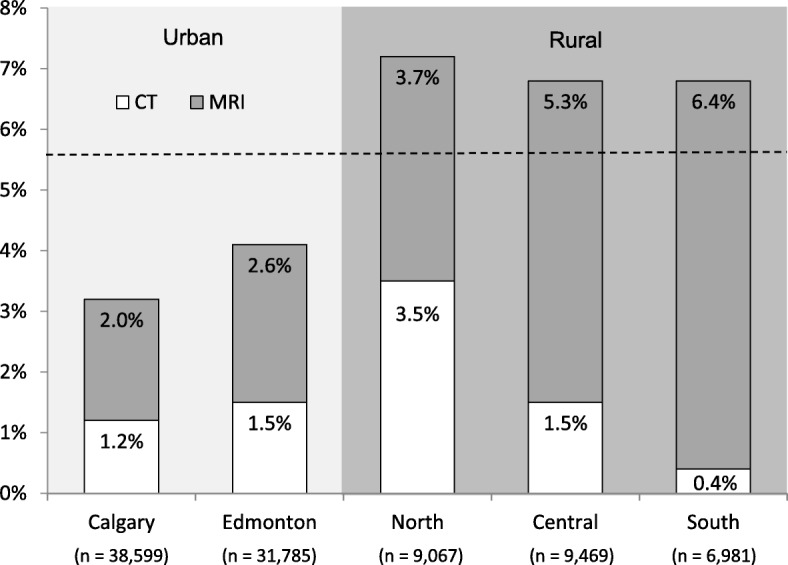
Risk-adjusted CT and MRI scan rates at 6 months after index visit for lower back pain by Albertan health zone

Estimates of overuse, adjusted for all significant factors in Table 1, are presented by Albertan health zone (Fig. 1). X-ray use was similar among the five zones. Use of CT/MRI demonstrated greater variability than X-ray testing, with the rural zones (North, Central, and South) displaying the highest rates of CT/MRI use. Calgary had the lowest CT/MRI rate at 3.2%, while the North had the highest rate at 7.2%. The apparent inverse relationship between number of lower back patients per health zone and CT/MRI imaging rates in Fig. 1 was not statistically significant, as assessed by a Spearman’s rank order correlation (*P* = .089).

### Preoperative cardiac testing

**Fig. 2:**
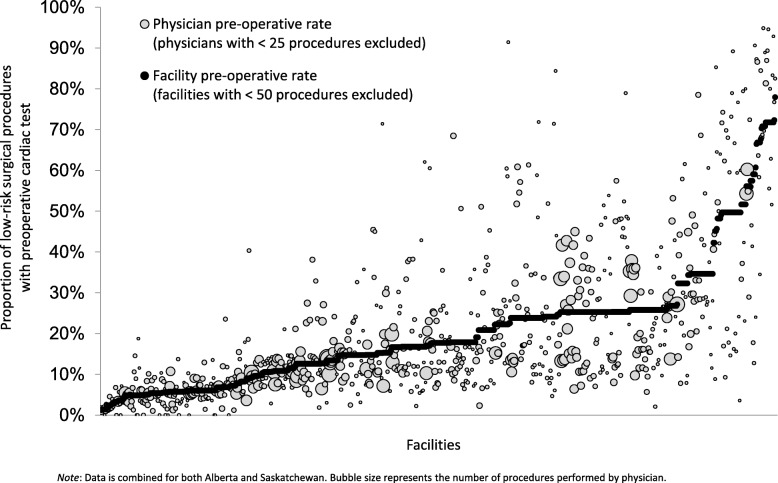
Physician- and facility-level variation in the proportion of low-risk surgical procedures with a preoperative cardiac test between June 2012 and March 2013 in Alberta and Saskatchewan

A total of 527,691 low-risk surgical procedures were observed during June 2012 and March 2013 (Ontario, *N* = 404,488; Alberta, *N* = 88,131; and Saskatchewan, *N* = 35,072). The proportion of low-risk surgeries with a preoperative cardiac test occurring within 60 days of the procedure was 17.9% in Alberta, 21.8% in Saskatchewan, and 35.5% in Ontario. Substantial facility- and physician-level variation in ordering was also observed (see Fig. 2).

Table 2 shows the adjusted associations of procedure-related factors with receipt of a preoperative cardiac test. Older age was found to be a statistically significant risk factor for a preoperative test in both Alberta (45–64 v 18–44, OR 3.40, *P* < 0.001; 65+ v 18–44, OR 4.89, *P* < 0.001) and Saskatchewan (45–64 v 18–44, OR 2.92, *P* < 0.001; 65+ v 18–44, OR 4.56, *P* < 0.001). Having either form of preoperative consultation compared with no consultation was strongly associated with having a preoperative cardiac test in either province.

**Table 2:** Patient- and provider-level characteristics and rates of preoperative cardiac testing within 60 days prior of a low-risk surgical procedure in Alberta (*N* = 88,131 procedures) and Saskatchewan (*N* = 35,072 procedures) between June 2012 and March 2013

			Preoperative cardiac testing	
Characteristic	Province^a^	No. procedures	Rate per 100^b^	Odds Ratio (95% CI)^c^
Patient-level**				
Age				
18–44 [ref]	Saskatchewan	5476	15.6	1.00
	Alberta	16,736	19.0	1.00
45–64	Saskatchewan	13,186	37.6	2.92 (2.63–3.23)*
	Alberta	33,759	38.3	3.40 (3.18–3.62)*
65+	Saskatchewan	16,410	46.8	4.56 (4.11–5.06)*
	Alberta	37,636	42.7	4.89 (4.56–5.24)*
Sex				
Male [ref]	Saskatchewan	16,134	46.0	1.00
	Alberta	40,945	46.5	1.00
Female	Saskatchewan	18,938	54.0	0.95 (0.90–1.00)
	Alberta	47,186	53.5	0.96 (0.93–1.00)
Comorbidity^d^				
No comorbidity [ref]	Saskatchewan	28,640	81.7	1.00
	Alberta	63,526	72.1	1.00
Comorbidity	Saskatchewan	6432	18.3	1.84 (1.73–1.96)*
	Alberta	24,605	27.9	1.90 (1.82–1.97)*
Provider-level***				
Anesthesiology consult				
No [ref]	Saskatchewan	34,077	97.2	1.00
	Alberta	85,055	96.5	1.00
Yes	Saskatchewan	995	2.8	6.16 (5.34–7.11)*
	Alberta	3076	3.5	10.9 (9.99–11.9)*
Medical consult				
No [ref]	Saskatchewan	32,432	92.5	1.00
	Alberta	82,150	93.2	1.00
Yes	Saskatchewan	2640	7.5	6.30 (5.78–6.76)*
	Alberta	5981	6.8	9.93 (9.36–10.5)*

Notes: * P ≤ .05; **patient characteristics are adjusted for each other and procedure type; *** provider characteristics are adjusted for each other and procedure type; CI = confidence interval; [ref] = reference category

^a^ separate models were created for each province versus including province as a dichotomous variable

^b^ rate is the observed rate (# tests/n) at index procedure – (1 to 60 d) for each patient in denominator

^c^ odds ratio and 95% CI estimates based on multiple binary logistic regression models and adjusted for procedure type (endoscopic, ophthalmologic, or other)

^d^ indicator variable representing whether patient had history of any of the following comorbidities: coronary artery disease, congestive heart failure, atrial fibrillation, other cardiac arrhythmia, cardiac valvular disease, renal disease, previous cardiovascular disease, peripheral vascular disease, venous thromboembolism, COPD, diabetes, hypertension and/or asthma

### Screening mammography

**Fig. 3:**
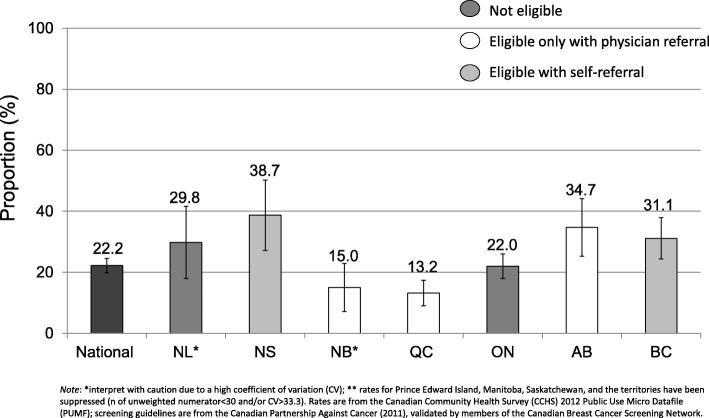
Proportion of Canadian women aged 40 to 49 who reported having a routine screening mammogram in the past two years despite being at average risk for breast cancer

After application of survey weights, there were 2,393,200 Canadian women aged 40–49 with valid responses to the 2012 CCHS mammography module. Of those respondents, 22.2% reported having at least one screening mammogram within the past two years, despite being of average-risk for breast cancer (weighted *n* = 532,300). This proportion was highly variable by province and breast cancer screening program eligibility for women aged 40–49 (Fig. 3). Provinces with self-referral (Nova Scotia and British Columbia) had among the highest rates of screening mammography (38.7 and 31.1% respectively). Despite the ineligibility of average-risk women aged 40–49 for public screening programs by either self or physician referral in select jurisdictions, mammography rates either approached or exceeded the national average in some provinces (Ontario 22.0%, Newfoundland and Labrador 29.8%).

The final respondent-level regression model is presented in Additional file 5: Table S5. Respondents who indicated they had a pap smear in the past three years had over a five-fold increase in the odds of having a screening mammogram versus respondents who did not report a pap smear in the same timeframe (OR 5.19, *P* < 0.001). Women who were presently or previously married had increased odds of having a mammogram versus single women (currently married, OR 1.64, *P* = 0.001; widowed/separated/divorced, OR 2.18, *P* < 0.001). Other statistically significant factors for having a routine mammogram include total annual household income above $59,999 (OR 1.37, *P* = 0.010) and having a regular medical doctor (OR 1.36, *P* = .048).

## Discussion

In our study, the first to measure unnecessary care across multiple Canadian provinces and territories, we found evidence of substantial overuse for each service we examined – lower back pain imaging in the absence of red flags, preoperative cardiac testing for low-risk surgeries, and routine screening mammography for women 40–49 years old – in scenarios where CWC recommendations indicate care is unnecessary or low-value [15–19]. Across these three non-recommended services, overuse ranged from 17.9 to 38.7% depending on the test and jurisdiction considered [21]. Moreover, our test-specific findings are consistent with previous smaller-scale studies in Canada [11, 12, 21]. As Kirkham et al. found in Ontario, our findings suggest preoperative cardiac testing is fairly common in Alberta and Saskatchewan (17.9 and 21.8% respectively) [10, 21]. Observed rates of CT/MRI scans for lower back pain in Alberta are similar to those reported by Pendrith and colleagues for Ontario [11]; however, the latter study investigated three-month rates [11, 21]. The national rate of screening mammography among women 40–49 was 22%, with significant variation among provinces. Our investigation revealed multiple factors associated with receipt of an unnecessary service. Older age, male sex, and living in a rural health zone were all patient characteristics associated with increased odds of lower back pain imaging. Patients with higher versus lower income had increased odds of having back imaging and early screening mammograms. Older, male patients have been previously found to be more likely to receive unnecessary testing have findings regarding older age and male previous studies that show that older age and male sex are patient characteristics are risk factors for potentially unnecessary electrocardiograms (ECGs) and chest x-rays [12, 25]. Contrary to our study, rural patients were less likely to receive a potentially unnecessary ECG, which differs from our findings for imaging for lower back pain and demonstrates the importance of understanding the specific drivers for individual unnecessary services [12]. All estimates were based on data that predates the 2014 launch of the CWC campaign and subsequent release of these three recommendations against low-value care; therefore, this study provides additional support to the existence of these recommendations [15–19, 21].

In 1998, the Institute of Medicine defined all healthcare quality problems as fitting into three categories: underuse, misuse, and overuse [34, 35]. Since then, much of the focus of research and quality improvement initiatives has been on underuse of appropriate medical services [36]. However, as concern has recently grown about the overprovision of medical services in situations where evidence does not support their use, so too has an interest in understanding how frequently inappropriate care is delivered [4, 5, 12]. The body of literature on overuse continues to grow, but to date, most of the research into overuse has been conducted in the U.S., with limited studies having been conducted in other countries, such as Sweden and Australia [5].

Overall estimates of overuse in Canada appear to be highly prevalent and remarkably similar to those in other countries, despite differences in healthcare systems and underlying populations [13, 37, 38]. However, comparing our test-specific findings with prior U.S. research demonstrates the need for country-specific data to understand which unnecessary services are potential drivers of overuse domestically [1]. For example, rates of imaging for lower back pain from the US ranged from 4.5 to 12.4% compared to our finding of 30.7% [6]. Based on data from two U.S. federally-sponsored health care programs (i.e. Veterans’ Affairs and Medicare), Kerr et al. concluded preoperative cardiac testing is a low priority issue after observing its low prevalence (0.62 to 4.34%) [6, 8]. Conversely, our Canadian study indicated low-value preoperative tests are relatively pervasive (17.9 to 35.5%) and worthy of further attention. These contrary findings show that we cannot simply generalize results from U.S. studies to other countries without careful consideration of the differences in the underlying patient cohorts and health care systems. Furthermore, this discrepancy emphasizes the importance of developing standardized definitions and measures of unnecessary care specific to a given country, and the need for international collaboration [8, 10, 14].

International disparities in the frequency of unnecessary care are unsurprising given the substantial variation we observed between and within levels of the Canadian health care system. For example, the rate of routine screening mammography among average-risk women aged 40–49 was nearly triple in Nova Scotia versus Quebec [21]; the rate of CT/MRI imaging was approximately two-fold in rural versus urban Albertan health regions [21]; and Albertan facilities ranged from almost never to nearly always ordering a cardiac test prior to a low-risk surgery [21]. This ordering variation across the Canadian health system is consistent with existing research and represents opportunities for provincial and local quality improvement initiatives to reduce unnecessary care by targeting those facilities and providers with above average ordering rates first [10–12, 14, 21].

This study represents the first attempt at measurement of unnecessary care at a national level both in Canada and internationally. However, several limitations with the study design are worth noting. While claims and survey data can help approximate the occurrence of unnecessary tests, these data sources lack clinical information that ultimately informs the care provider’s decision to recommend, order, or withhold a service [14]. We captured imaging at six months to achieve comparability with prior research while also considering wait times in Alberta (e.g. MRI 90th percentile wait time, 235 days) [11, 21, 23, 24]. Consequently, some imaging may have been indicated for cases of chronic back pain or other clinical conditions with lumbar pain symptoms. Responses to the CCHS are voluntary and self-reported, and as such, may be subject to volunteer and social desirability biases [28, 29]. Furthermore, cancer screening rates derived from CCHS data are consistently higher than rates found using administrative data [39]. Additionally, there are several limitations that highlight the challenges faced when estimating overuse as a national population level and the importance of common methodological approaches to quantifying low-value care. Jurisdictional variation may be partially attributed to the lack of standardized billing codes and public health programs, as well as differential access to diagnostic services [21]. To achieve comparisons between Alberta and Ontario, we identified lower back pain diagnoses using three-digit ICD-9 codes, which may have overestimated the number of patients compared to using more specific four- or five-digit codes [21]. However, among Albertan visits where fourth and fifth digits were available, we found that 80% of the visits selected using three-digit codes were for lower-back pain [21]. Finally, the regression analyses were not standardized across the three measures studied. While the variables examined were informed by previous studies, due to inconsistencies in the data sets used, we were unable to use a standard set of variables for each regression model.

## Conclusions

Despite the limitations, this is the first study to provide large-scale estimates of unnecessary care across multiple Canadian provinces. Overall, the prevalence of unnecessary care in Canada appears similar to the U.S. despite differences in the frequency of individual tests. Moreover, there was striking inter- and intra-provincial variation in unnecessary care ordering frequency across Canada. We identified several factors associated with multiple examples of inappropriate care (i.e. patient age and income), which may provide insight into ordering variation that can assist the development of strategies undertaken to promote resource stewardship. Subsequent research should explore associations between interventions to decrease the frequency of unnecessary care, downstream testing, and patient outcomes.

## Additional files

Additional file 1: **Table S1.** Utilization of unnecessary services related to three Choosing Wisely Canada (CWC) recommendations. Table describing CWC recommendations for lower back pain imaging, preoperative cardiac testing, and screening mammography, and methodological details pertaining to each of the three studies. (DOCX 16 kb)

Additional file 2: **Table S2.** Lower back pain imaging methodology summary. Tables describing methodological details and relevant codes used for lower back pain study. (DOCX 16 kb)

Additional file 3: **Table S3.** Preoperative cardiac testing methodology summary. Tables describing methodological details and relevant codes used for preoperative cardiac testing study. (DOCX 17 kb)

Additional file 4: **Table S4.** Screening mammography methodology summary. Tables describing methodological details and data handling procedure for screening mammography study. (DOCX 15 kb)

Additional file 5: **Table S5.** Screening mammography respondent-level regression model results. Association between respondent characteristics and reporting a screening mammogram in the past two years for Canadian women aged 40–49 at average-risk for breast cancer – weighted. (DOCX 13 kb)

## Data Availability

The data that support the findings of this study are available from the Canadian Institute of Health Information, but legislative restrictions apply to the availability of these data, are not publicly available. The authors obtained permissions to access the relevant data for this study through a formal request to CIHI as part of a collaborative research effort.

## Abbreviations

CCHS: Canadian Community Health Survey
CCI: Canadian Classification of Health Interventions
CIHI: Canadian Institute for Health Information
CT: Computed tomography
CW: Choosing Wisely
CWC: Choosing Wisely Canada
DAD: Discharge Abstract Database
ECG: Electrocardiogram
ICD: International Classification of Diseases
ICES: Institute for Clinical Evaluative Sciences
IOM: Institute of Medicine
MRI: Magnetic resonance imaging
NACRS: National Ambulatory Care Reporting System
OR: Odds ratio
PLPB: Patient-Level Physician Billing
TTE: Transthoracic echocardiogram
