# Supplementary material for: Measuring the frequency and variation of unnecessary care across Canada
Source: BMC Health Serv Res. 2019 Jul 3;19:446. doi: 10.1186/s12913-019-4277-9 (PMC6610789; doi:10.1186/s12913-019-4277-9)
Supplement: Supplementary file 1 — Table S1. Utilization of unnecessary services related to three Choosing Wisely Canada (CWC) recommendations. Table describing CWC recommendations for lower back pain imaging, preoperative cardiac testing, and screening mammography, and methodological details pertaining to each of the three studies. (DOCX 16 kb) [file 12913_2019_4277_MOESM1_ESM.docx]

**Additional file 1:Table S1 – Utilization of unnecessary services related to three Choosing Wisely Canada (CWC) recommendations.**

| **Description** | **Rationale** | **Data sources** | **Denominator** | **Numerator(s)** |
| --- | --- | --- | --- | --- |
| 1. Don’t do imaging for lower back pain unless red flags are present (CAR, CFPC)  **Date of recommendation:** April 2^nd^, 2014  **Study design:** retrospective cohort | Red flags include suspected epidural abscess or hematoma presenting with acute pain, but no neurological symptoms; suspected cancer; suspected infection; cauda equina syndrome; severe or progressive neurologic deficit; and suspected compression fracture. In patients with suspected uncomplicated herniated disc or spinal stenosis, imaging is only indicated after at least a six-week trial of conservative management and if symptoms are severe enough that surgery is being considered.  Imaging of lower back pain is not recommended for several reasons. First, acute lower back pain typically resolves without treatment within 4 weeks. Furthermore, imaging results may not be sufficiently informative to influence treatment plans because most lumbar imaging abnormalities are not specifically related to lower back pain symptoms. | PLPB, DAD, NACRS | Adult patients (≥18 years old) in Alberta who sought help from a family physician for non-persistent lower back pain between April 1^st^, 2011 and March 31^st^, 2012. Excluded patients and facilities not residing in Alberta, patients with persistent lower back pain or with a history of lower back pain, related imaging or surgery, or other red flags within 12 months prior to the index visit. | **For X-rays:** Individuals from denominator with ≥ 1 spinal X-ray in NACRS or PLPB claims data within 3, 6, and 12 months following the date of the index visit.  **For CT/MRI:** Individuals from denominator with ≥ 1 CT and/or MRI in NACRS claims data within 3, 6 and 12 months following the date of the index visit. |
| 2. Don’t routinely perform preoperative testing for patients undergoing low-risk surgeries (CAS, CCS, CSIM)  **Date of recommendation:** April 2^nd^, 2014  **Study design:** retrospective cohort | Routine preoperative tests for low-risk surgeries results in unnecessary delays, potential distress for patients and significant cost for the health care system. Numerous studies and guidelines outline lack of evidence for benefit in routine preoperative testing (e.g. chest X-ray, echocardiogram) in low-risk surgical patients. | PLPB, DAD, NACRS, ICES/CWC^a^ | Surgical procedures conducted on adult patients (≥ 18 years old) deemed to be low-risk (< 1% estimated risk of myocardial infarction or cardiac-related death). Included procedures performed in acute care (on the same date as admission) or within an ambulatory care setting between June 1^st^, 2012 and March 31^st^, 2013 in Saskatchewan, Alberta, or Ontario. | Procedures from denominator associated with ≥ 1 claim for a cardiac test within 60 days of the procedure (index event). Cardiac tests included: a) electrocardiogram, b) echocardiography, c) chest X-ray, and d) cardiac stress tests.  Claims for the above tests were not counted if they occurred on the same day as surgery as they may have been postoperative. |
| 3. Don’t do screening mammography for average-risk women aged 40-49 (CFPC).  **Date of recommendation:** October 29^th^, 2014^c^  **Study design:** cross-sectional | A 2011 recommendation from the CTFPHC suggests average-risk women wait until age 50 before initiating regular breast cancer screening.  After careful assessment of women less than 50 years of age, if their risk profile for breast cancer risk is not high, the benefit of screening mammography is small. Furthermore, this age group has a greater risk of false-positive screening results and consequently of undergoing unnecessary or harmful follow-up procedures. | CCHS | Canadian women aged 40-49 years who responded to the 2012 CCHS, excluding proxy interviews. | Individuals from the denominator who indicated they had a mammogram within the last 2 years preceding the survey and also indicated an average-risk reason (‘part of regular check-up/routine screening’ and/or ‘age’) for having the mammogram^b^.  Respondents selecting other options for reason for mammogram listed below, were excluded from the numerator and deemed high-risk:  -Family history of breast cancer  -Previously detected lump  -Follow-up of breast cancer treatment  -On hormone replacement therapy  -Breast problem  -Other |
| *Notes:* CAR = Canadian Association of Radiologists; CFPC = College of Family Physicians of Canada; CAR = Canadian Anesthesiologists’ Society; CSIM = Canadian Society of Internal Medicine; PLPB = Patient-Level Physician Billing; DAD = Discharge Abstract Database; NACRS = National Ambulatory Care Reporting System; ICES = Institute for Clinical Evaluative Sciences; CTFPHC = Canadian Task Force for Preventative Health Care; CCHS = Canadian Community Health Survey.  ^a^ ICES and CWC shared aggregated data for Ontario from prior study.  ^b^ average-risk definition based on input from the CWC Family Medicine expert group.  ^c^ recommendation originally released on October 29^th^, 2011 but was updated in summer 2016 to reflect patient preferences. | | | | |
