# Supplementary material for: Measuring the frequency and variation of unnecessary care across Canada
Source: BMC Health Serv Res. 2019 Jul 3;19:446. doi: 10.1186/s12913-019-4277-9 (PMC6610789; doi:10.1186/s12913-019-4277-9)
Supplement: Supplementary file 2 — Table S2. Lower back pain imaging methodology summary. Tables describing methodological details and relevant codes used for lower back pain study. (DOCX 16 kb) [file 12913_2019_4277_MOESM2_ESM.docx]

**Additional file 2: Table S2 – Lower back pain imaging methodology summary.**

| **Index Cohort** | |
| --- | --- |
| Data source | - Patient Level Physician Billing Data Repository (PLPB) - Discharge Abstract Database (DAD) - National Ambulatory Care Reporting System (NACRS) |
| Cohort | - Adult patients (age 18+) with non-persistent lower back pain who sought medical help from family physicians in Alberta between April 1, 2011 to March 31, 2012 - Only the first family physician visit for lower back pain was included for each patient |
| Index event exclusions | - Patients with persistent lower back pain who had a history of lower back pain, related imaging or surgery, or red flags within a year prior to the index visit were excluded. These conditions were identified using billing data, emergency department visits or acute care hospitalizations |
| **Outcomes** | |
| Outcome of interest | - X-rays in ambulatory or day care facilities, or in the community within 3, 6 and 12 months after the index visit - Combined measure of CT and/or MRI scans in ambulatory or day care facilities within 3, 6, and 12 months after the index visit - 6-month scan rates were used for predictor analysis, and regression modelling was used to derive adjusted odds ratios |
| Data source | - X-rays were identified from NACRS and PLPB - CT and MRI scans were identified from NACRS |

**Lower back pain case inclusion codes.**

| ICD-9 (PLPB)  *Used for index event and prior history* | ICD-10-CA (NACRS and DAD)  *Only used for prior history of lower back pain* |
| --- | --- |
| 721, 722, 724, 847, 846 | M47.86, M47.87, M47.88, M48.96, M48.97, M47.96, M47.97, M47.98, M51.1, M51.2, M51.3, M51.9, M53.86, M53.87, M53.88, M48.06, M48.07, M46.36, M46.37, M46.46, M46.47, M54.5, M54.3, M54.4, M54.8, M54.9, M43.27, M43.28, M53.27, M53.28, M53.3, M43.9, M43.96, M43.97, M43.98, M99.83, M99.93, M99.03, M99.84, M99.94, M99.04, S33.5, M53.26, M53.27, M53.28, S33.5, S33.6, S33.7 |

**Lower back pain exclusions.**

1. Patient with health card not issued by Alberta
2. Submitting facility is not in Alberta
3. Invalid health card numbers
4. encounters in acute care (PLPB)
5. Age < 18 years on date of visit
6. Visits to diagnostic labs or imaging facility
7. Prior diagnosis of lower back pain (LBP) – 1 to 365 days prior to LBP diagnosis (PLPB = ICD-9 codes; NACRS and DAD = ICD-10-CA codes)
8. Previous visits to neuro-surgeons or ortho-surgeons; or for spine surgeries in the previous 12 months (1-365 days)

- Visits to neuro-surgeons or ortho-surgeons (PLPB):
  - Neuro-surgeon or ortho-surgeon visits (specialty for the claim: 280, 335)
  - billing code starting with “16”
- Spine surgeries (NACRS and DAD CCI codes):
  1.AW.^^.^^, 1.SC.^^.^^, 1.SE.^^.^^, 1.SF.^^.^^, 1.SG.^^.^^, 1.SH.^^.^^, 1.SI.^^.^^, 1.SJ.^^.^^

1. Prior diagnostic imaging of the spine – 1 to 365 days prior to LBP diagnosis. See Appendix 3 for billing and CCI codes.
2. Red flag exclusion criteria: Cancer, neurological problem, specific infections, vertebral compression fracture – 0 to 365 days prior to LBP diagnosis (PLPB ICD-9 codes, NACRS ICD-10-CA codes):

| **Red flag category** | **ICD-9 (PLPB)** | **ICD-10-CA (NACRS and DAD)** |
| --- | --- | --- |
| Cancer / history of cancer | 140-208, 230-239, V10, V580, V581 | C00-C97, D00-D09, D37-D48, Z51.0, Z51.1, Z85, Z86 |
| Neurological problems | 728, 781, 787, 788, 345, 331, 353, 333, 340, 332, 357, 351, 350, 349, 348, 344, 337, 358, 323, 359, 341, 342, 334 | M62.9, R56, R29.8, R15, R32, G40, G30, G31, G32, G20-G26, G35, G96.1, G96.8, G96.9, G97, G98, G93, G82, G83, G90, G04, G05, G37, G81, G11, G54, G61, G62.0, G62.1, G62.2, G51, G50, G70, G71, G72 |
| Specific infections / fever **3 months prior to back pain visit** | 010-018, 038, 730, 997, 998, 720 | A15-A19, A40, A41, M86, M46.2, M89.6, T87.4, T81.4, G06.2, G06.1, M46.3, M46.5 |
| Vertebral compression fracture | 733 | M84.48, M90.7,  (M80.0 to M80.9 with a 5th digit of “8”) |

**Lower back pain imaging codes.**

*Note:* Imaging tests identified from AB’s billing data and NACRS- 0-365 days following LBP index visit

| Type of Diagnostic Imaging | PLPB (billing codes) | NACRS (CCI codes) |
| --- | --- | --- |
| X-ray | X55, X56, X57, X57A, X58E, X58, X59, X60, X61, X62, X63, X64, X65, X66, X67 | 3.SC.10.^^ , 3.SE.10.^^, 3.SF.10.^^  3.SC.12.^^, 3.SE.12.^^, 3.SF.12.^^ |
| CT | N/A | 3.SC.18.^^, 3.SF.18.^^  3.SC.20.^^, 3.SF.20.^^ |
| MRI | N/A | 3.SC.40.^^, 3.SF.40.^^ |
