# Supplementary material for: Measuring the frequency and variation of unnecessary care across Canada
Source: BMC Health Serv Res. 2019 Jul 3;19:446. doi: 10.1186/s12913-019-4277-9 (PMC6610789; doi:10.1186/s12913-019-4277-9)
Supplement: Supplementary file 3 — Table S3. Preoperative cardiac testing methodology summary. Tables describing methodological details and relevant codes used for preoperative cardiac testing study. (DOCX 17 kb) [file 12913_2019_4277_MOESM3_ESM.docx]

**Additional file 3: Table S3 - Preoperative cardiac testing methodology summary.**

| **Low-Risk Procedures** | |
| --- | --- |
| Data source | - ICES/CWC provided aggregate data for Ontario - Alberta and Saskatchewan   - Discharge Abstract Database (DAD)   - National Ambulatory Care Reporting System (NACRS) |
| Low-risk procedure inclusions | - Low-risk procedures were identified by an expert panel (endoscopy, ophthalmology and other – see appendix 1 for full list) - Discharges from ambulatory/day and acute care facilities from June 2012 to March 2013 - Low-risk was identified as procedures in acute care on the same day as admission (DAD) or performed in an ambulatory care setting (NACRS/DAD) - Principle intervention code (DAD) or first listed intervention (NACRS) were used. |
| Low-risk procedure exclusions | - Records with invalid healthcare numbers or gender - Procedures performed after the first day of admission (even if admission was elective) - Duplicate procedures based on health care number and issuing province, date and procedure type - Patients aged <18 years - Procedures in facilities with <50 low-risk procedures performed (this exclusion takes out facilities with potentially unstable rates due to low volumes) |
| **Preoperative Testing** | |
| Outcome of interest | - Presence of cardiac testing (described below) 1-60 days prior to a low-risk procedure. |
| Data source | - ICES/CWC provided aggregate data for Ontario - Alberta and Saskatchewan   - Patient Level Physician Billing data (PLPB)   - Discharge Abstract Database (DAD)   - National Ambulatory Care Reporting System (NACRS) |
| Preoperative testing inclusions | - Cardiac tests in Alberta and Saskatchewan occurring in acute or ambulatory (day) care facilities, or in the community were included. (See appendix 2 for list of codes used to identify cardiac testing: ECG, cardiac stress testing, echocardiography and chest x-ray) - NOTE: Testing may or may not be reported to DAD/NACRS. For example, tests done in the community will appear only in the PLPB, tests done in hospital may also appear in PLPB but may or may not appear in DAD/NACRS. If cases are reported/captured in duplicate, only one test was included. |
| Preoperative testing exclusions | - Duplicates based on health care number and issuing province, date and test type (ECG, echocardiography, x-ray, stress test) - Cardiac testing performed on the same day as surgery was deemed non-screening and may have been performed post-procedure |

**Low-risk surgical procedure ICD-10 codes.**

| **Endoscopy**  Esophagus/Stomach   - 2.NA.70.BA, 2.NA.71.BA, 2.NA.71.NP, 2.NA.71.BR, 2.NC.70.BA, 2.NF.70.BA, 2.NF.71.BA, 2.NF.71.BP, 2.NF.71.BR   Large Bowel   - 2.NM.70.BA, 2.NM.71.BA, 2.NM.71.BR |
| --- |
| **Ophthalmology**  *Other Ophthalmology*   - 1.CC, 1.CD, 1.CE, 1.CF, 1.CG, 1.CH, 1.CJ, 1.CL, 1.CM, 1.CN, 1.CP, 1.CQ, 1.CR, 1.CS, 1.CT, 1.CU, 1.CV, 1.CX, 1.CZ   Secondary Cataract   - 1.CL.59   Cataract   - 1.CL.89 |
| **Other**  *Orthopedic*  Shoulder (endoscopic drainage/extraction/procurement/release)   - 1.TA.52.DA, 1.TA.57.DA, 1.TA.58.DA, 1.TA.72.DA, 1.TA.80.DA (All sub codes), 1.TA.80.GZ   Clavicle (endoscopic drainage/distal resection)   - 1.TB.52.GB, 1.TB.52.GD, 1.TB.87.DA   Rotator Cuff (endoscopic extraction/release/repair)   - 1.TC.57.DA, 1.TC.59.DA 1.TC.72.DA, 1.TC.80.DA (all sub codes) 1.TC.80.GC (all sub codes)   Arm/Forearm (Nerve decompression/repair/excision)   - 1.BM.72, 1.BM.80, 1.BM.87, 1.BN.72   Wrist/Hand   - 1.UB.52, 1.UB.53, 1.UB.55, 1.UB.57, 1.UB.58, 1.UB.72, 1.UB.73, 1.UB.74, 1.UB.75, 1.UB.80, 1.UB.87, 1.UC.53, 1.UC.55, 1.UC.57, 1.UC.72, 1.UC.73, 1.UC.74, 1.UC.75, 1.UC.79, 1.UC.80, 1.UC.82, 1.UC.87, 1.UC.89, 1.UF.55, 1.UF.73, 1.UF.74, 1.UF.80, 1.UF.87, 1.UG.52, 1.UG.53, 1.UG.55, 1.UG.57, 1.UG.72, 1.UG.73, 1.UG.74, 1.UG.75, 1.UG.80, 1.UG.87, 1.UJ.71, 1.UJ.73, 1.UJ.74, 1.UJ.75, 1.UJ.82, 1.UJ.87, 1.UJ.93, 1.UK.53, 1.UK.55, 1.UK.72, 1.UK.73, 1.UK.74, 1.UK.75, 1.UK.80, 1.UK.87, 1.UK.93, 1.US.58, 1.US.72, 1.US.80, 1.UT.53, 1.UT.55, 1.UT.72, 1.UT.80, 1.UT.84, 1.UU.53, 1.UU.55, 1.UU.72, 1.UU.80, 1.UU.84, 1.UV.72, 1.UV.80, 1.UY.52, 1.UY.55, 1.UY.56, 1.UY.57, 1.UY.59, 1.UY.72, 1.UY.80, 1.UY.87   Nerve   - 1.BP.72, 1.BP.80, 1.BP.87, 1.BQ.72, 1.BQ.80, 1.BQ.87   Hip Arthroscopy (extraction/procurement/release/partial excision)   - 1.VA.57.DA, 1.VA.58.DA, 1.VA.72.DA, 1.VA.87.DA, 1.VA.87.GB   Knee  Arthroscopy (drainage/extraction/procurement/release/partial excision)   - 1.VG.52.DA, 1.VG.57.DA, 1.VG.58.DA, 1.VG.72.DA, 1.VG.87.DA, 1.VG.87.GB   Meniscus (endoscopic repair/partial or total excision)   - 1.VK.80.DA (all sub codes), 1.VK.87.DA, 1.VK.89.DA   Ligament (ACL) (endoscopic repair/partial excision)   - 1.VL.80.DA, 1.VL.80.FY, 1.VL.87.DA, 1.VL.87.GB   Ankle/Foot Arthroscopy (extraction/procurement/release)   - 1.WA.57.DA, 1.WA.58.DA, 1.WA.72.DA   Microdiscectomy   - 1.SE.87   *Urologic*  Bladder neck suspension   - 1.PL.74   Transurethral partial excision   - 1.PL.87   Bladder Drainage   - 1.PM.52, 1.PM.54   Bladder   - 1.PM.59   Prostate resection (TURP)   - 1.QT.87   Urethra   - 1.PQ.26, 1.PQ.35, 1.PQ.50, 1.PQ.52, 1.PQ.53, 1.PQ.54, 1.PQ.55, 1.PQ.57, 1.PQ.58, 1.PQ.59, 1.PQ.72, 1.PQ.77, 1.PQ.78, 1.PQ.80   *Gynecologic*  Hysteroscopy (endometrial ablation)   - 1.RM.59.BA   Laparoscopy (oophorectomy, cystectomy)   - 1.RB.52.BA, 1.RB.52.DA, 1.RB.56.DA, 1.RB.74.DA, 1.RB.87.DA, 1.RB.89.DA, 1.RD.52.BA, 1.RD.89.DA   *General*  Hernia repair (repair muscles of chest and abdomen)   - 1.SY.80   Inguinal lymph nodes   - 1.MJ.52, 1.MJ.87, 1.MJ.89   Peripheral lymph nodes   - 1.MK.52, 1.MK.87, 1.MK.89   Breast (removal of device/fixation/size reduction/size increase/repair/partial or total excision/   - 1.YM.55, 1.YM.74, 1.YM.78, 1.YM.79, 1.YM.80, 1.YM.87, 1.YM.89   Laparoscopic Cholecystectomy   - 1.OD.57 |

**Endoscopy codes in PLPB.**

| Source | Codes |
| --- | --- |
| SK PLPB | L402, L408, L360, L448, L449, L450, L492, L529 |
| AB PLPB | 1.12, 01.12A, 01.14, 01.22, 01.22A, 01.22B, 01.22C, 01.24A, 01.24B, 01.24BA, 01.24BB |

**Preoperative cardiac testing codes.**

| Source | ECG | Echocardiogram | Stress test | Chest x-ray |
| --- | --- | --- | --- | --- |
| DAD/NACRS (CCI) | 2.HZ.24^ | 3.IP.30^ | 2.HZ.08^ | 3.IK.10^, 3.IM.10^, 3.IN.10^, 3.IP1.0^, 3.IS.10^ |
| SK PLPB | D030, D031, D032 | A320, A520, A530, A321, A521, A531, A322, A522, A532, A556, A523, A533, A557, W020, A323  A324, A534 | D62, D63, D64, D65, D66, D67 | X150, X158, X159 |
| AB PLPB | 03.52A, 03.52B | X306, X307 | X170, X171, X172, X173 03.41A, 03.41B, 03.41C, 03.41D, 03.44A | X 20, X 20A, X 20B, X 21*  *spaces intentional |
