# Supplementary material for: Measuring the frequency and variation of unnecessary care across Canada
Source: BMC Health Serv Res. 2019 Jul 3;19:446. doi: 10.1186/s12913-019-4277-9 (PMC6610789; doi:10.1186/s12913-019-4277-9)
Supplement: Supplementary file 4 — Table S4. Screening mammography methodology summary. Tables describing methodological details and data handling procedure for screening mammography study. (DOCX 15 kb) [file 12913_2019_4277_MOESM4_ESM.docx]

**Additional file 4: Table S4 - Screening mammography methodology summary.**

| **Index Cohort** | |
| --- | --- |
| Data source | - 2012 Canadian Community Health Survey (CCHS) |
| Sample | - Female respondents aged 40 to 49 years from the CCHS |
| **Outcomes** | |
| Outcome of interest | The number of average risk women aged 40 to 49 years who reported having a screening mammogram in the past 2 years, based on responses to “MAM_030: Have you ever had a mammogram, that is, a breast x-ray?” and “MAM_032: When was the last time?”  Risk status was defined based on respondents’ answers for the reason for the mammogram, “MAM_31: Why did you have it?”. Respondents could select all response options that applied.  • Average risk was limited to respondents who selected either or both of the following reasons for having a mammogram:  -Part of regular check-up/ routine screening  -Age  • Respondents were excluded if they indicated any of the other reasons for a mammogram, listed below:  -Family history of breast cancer  -Previously detected lump  -Follow-up of breast cancer treatment  -On hormone replacement therapy  -Breast problem  -Other  • Note that this definition was developed by CIHI with input from the CWC family medicine group. |
| Data source | - 2012 CCHS |

**Data handling procedure (as per CCHS Public Use Microdata File (PUMF) data release rules, 2012).**

| **Rounding** | Estimates or volumes: rounded to the nearest hundred.  Percentages and proportions: rounded to one decimal place and calculated from unrounded numerators and denominators.  The rounding is necessary because unrounded estimates would imply greater precision than what exists. |
| --- | --- |
| **Weighting** | Survey weights were applied to be representative of the population (variable WTS_M) |
| **Data Suppression** | • For releasing weighted data, if the number of unweighted respondents (n) is less than 30, the weighted estimate cannot be released. If (n) is greater than or equal to 30, the coefficient of variation (CV) determines whether or not the data can be released:   - If 0.0≤CV≤16.5 then weighted data can be released  - If 16.6≤CV≤33.3 then weighted data can only be released if a warning or caution symbol accompanies the estimate, notifying the user of the large sampling variability for the estimate  - If the CV>33.3, the estimate cannot be released • Coefficients of variation were determined using approximate sampling variability look-up tables provided by Statistics Canada  • Further information on the release of CCHS data from the PUMF can be found in the 2012 CCHS User Guide, available by request from Statistics Canada. |
| **95% Confidence Intervals** | • 95% confidence intervals were calculated using coefficients of variation found in approximate sampling variability look-up tables provided by Statistics Canada.  • A limitation of these 95% confidence intervals is that they are based on approximate coefficients of variation, and therefore may not be exact. |
