# Supplementary material for: Measuring the frequency and variation of unnecessary care across Canada
Source: BMC Health Serv Res. 2019 Jul 3;19:446. doi: 10.1186/s12913-019-4277-9 (PMC6610789; doi:10.1186/s12913-019-4277-9)
Supplement: Supplementary file 5 — Table S5. Screening mammography respondent-level regression model results. Association between respondent characteristics and reporting a screening mammogram in the past two years for Canadian women aged 40–49 at average-risk for breast cancer – weighted. (DOCX 13 kb) [file 12913_2019_4277_MOESM5_ESM.docx]

**Additional file 5: Table S5 - Screening mammography respondent-level regression model results.**

| **Exhibit 5.** Association between respondent characteristics and reporting a screening mammogram in the past two years for Canadian women aged 40-49 at average-risk for breast cancer – weighted N = 2,393,200. | | |
| --- | --- | --- |
| Factor | | Odds Ratio (95% CI) |
| Pap smear, past 3 y** |  | |
| Yes v no | 5.19 (3.93-6.85)* | |
|  |  | |
| Cultural or racial origin** |  | |
| Visible minority v white | 2.53 (2.08-3.06)* | |
|  |  | |
| Marital status |  | |
| Married or common law v single | 1.64 (1.22-2.22)* | |
| Widowed/separated/divorced v single | 2.18 (1.54-3.09)* | |
|  |  | |
| Physical activity |  | |
| Moderately active v active | 1.64 (1.30-2.06)* | |
| Inactive v active | 1.11 (0.89-1.38) | |
|  |  | |
| Total household income (all sources) |  | |
| $60,000 or more v less than $60,000 | 1.37 (1.08-1.74)* | |
|  |  | |
| Regular medical doctor |  | |
| Yes v no | 1.36 (1.00-1.84)*^,a^ | |
|  |  | |
| BMI group** |  | |
| Underweight v normal weight | 0.36 (0.19-0.67)* | |
| Overweight v normal weight | 0.92 (0.74-1.13) | |
| Obese v normal weight | 0.84 (0.66-1.07) | |
|  |  | |
| Jurisdictional screening eligibility |  | |
| Not eligible v self-referral | 0.51 (0.41-0.64)* | |
| Physician referral v self-referral | 0.55 (0.43-0.70)* | |
| *Note*: CI = confidence interval; *significant at *p* < .05; **comparisons between non-response levels and reference categories for applicable variables not reported.  ^a^ *p*-value = .048. | | |
